# Task Offloading and Resource Allocation for ICVs in Vehicular Edge Computing Networks Based on Hybrid Hierarchical Deep Reinforcement Learning

**DOI:** 10.3390/s25226914

**Published:** 2025-11-12

**Authors:** Jiahui Liu, Yuan Zou, Guodong Du, Xudong Zhang, Jinming Wu

**Affiliations:** 1 School of Mechanical Engineering, Beijing Institute of Technology, Beijing 100081, China; 3120205209@bit.edu.cn (J.L.); xudong.zhang@bit.edu.cn (X.Z.); 3120205222@bit.edu.cn (J.W.); 2 National Engineering Research Center for Electric Vehicles, Beijing Institute of Technology, Beijing 100081, China; 3 Beijing Institute of Technology Chongqing Innovation Center, Chongqing 401100, China

**Keywords:** vehicular edge computing networks, mobile edge computing, task offloading, resource allocation, deep reinforcement learning

## Abstract

Intelligent connected vehicles (ICVs) face challenges in handling intensive onboard computational tasks due to limited computing capacity. Vehicular edge computing networks (VECNs) offer a promising solution by enabling ICVs to offload tasks to mobile edge computing (MEC), alleviating computational load. As transportation systems are dynamic, vehicular tasks and MEC capacities vary over time, making efficient task offloading and resource allocation crucial. We explored a vehicle–road collaborative edge computing network and formulated the task offloading scheduling and resource allocation problem to minimize the sum of time and energy costs. To address the mixed nature of discrete and continuous decision variables and reduce computational complexity, we propose a hybrid hierarchical deep reinforcement learning (HHDRL) algorithm, structured in two layers. The upper layer of HHDRL enhances the double deep Q-network (DDQN) with a self-attention mechanism to improve feature correlation learning and generates discrete actions (communication decisions), while the lower layer employs deep deterministic policy gradient (DDPG) to produce continuous actions (power control, task offloading, and resource allocation decision). This hybrid design enables efficient decomposition of complex action spaces and improves adaptability in dynamic environments. Results from numerical simulations reveal that HHDRL achieves a significant reduction in total computational cost relative to current benchmark algorithms. Furthermore, the robustness of HHDRL to varying environmental conditions was confirmed by uniformly designing random numbers within a specified range for certain simulation parameters.

## 1. Introduction

Admidst the swift advancement of Internet of Things (IoT) technology, the vehicle–road–cloud integration system stands as an inexorable trajectory in the progression of future intelligent transportation systems, which organically links pedestrians, vehicles, roads, clouds, and other transportation participation elements together [1]. As an important participant in the dynamic transportation system, the ICV is responsible for performing important functions such as application tasks and interacting with the road/cloud. However, with the increase in computation-intensive in-vehicle applications and sensor data, the burden of the task calculation for the ICV in-vehicle computing platform has become heavier. These tasks require high-frequency processing and higher energy consumption. Limited by hardware and cost, the in-vehicle computing platform is challenged by completing its work with quality and quantity within the demanded time. To address this challenge, offloading vehicular tasks to MEC servers within roadside units (RSUs) through vehicular edge computing networks (VECNs) is considered an effective solution. The unit of MEC serves as a new small data storage and processing center, effectively connecting the vehicle terminal (VT), road terminals, and the cloud. It offers advantages such as rapid responsiveness, high processing efficiency, minimal network pressure, strong data security, and so on [2].

Relying on vehicular network technology and 5G network technology, the calculation task of VTs can be offloaded and completed on the MEC servers, which effectively alleviates the pressure on VTs caused by limited computing and storage resources, while significantly reducing task execution latency and energy consumption [3]. In real-world scenarios, MEC servers are positioned at the edge of vehicular networks, allowing ICVs to access them through communication networks via base stations or roadside access points. However, the application tasks are dynamic and have different latency thresholds, and the MEC server’s available computing capacity fluctuates over time. Therefore, choosing the appropriate task offloading and computational mechanism to achieve smooth computational completion of VT and MEC servers [4] is the main challenge. It is of great importance to dynamically choose the MEC servers, determine the percentage of tasks offloaded to them, and simultaneously consider other factors such as vehicle status and communication delays. At the same time, it is well known that most MEC servers are underutilized, operating only at 10–30% capacity [5]. This also reminds us that reasonable offloading tasks, ensuring the completion of tasks, and energy saving are meaningful for ICVs to improve their performance through optimizing the utilization of VT and MEC computation capacity.

To adapt to environmental changes and reduce the comprehensive costs of time and resource scheduling in VECNs, we design an optimization problem that considers long-term comprehensive costs. We then propose an HHDRL algorithm framework, based on deep reinforcement learning (DRL), to tackle the problem. The goal is to minimize the total long-term cost of multi-vehicle task scheduling and offloading, accounting for both time delays and energy consumption. And the parameter settings differ from those in traditional simulations. Instead of using fixed values, we generate parameters uniformly and randomly within a predetermined range to better simulate the changes in system parameters observed in real-life scenarios. The key contributions of the paper are outlined as follows:

- A vehicle–road collaborative edge computing network is proposed for efficient task offloading, where tasks from ICVs can be processed locally or offloaded to MEC for collaborative processing. The task offloading scheduling and resource allocation (TOSRA) optimization problem is formulated to effectively minimize the overall time and energy consumption costs of the dynamic system.
- To address the problem, TOSRA is decomposed into two-layer subproblems, and an HHDRL algorithm is proposed to achieve the optimal solution. The upper layer incorporates an SA-DDQN algorithm with a self-attention mechanism for generating communication decisions (CDs), and the lower layer applies a deep deterministic policy gradient (DDPG) algorithm to manage power control, task offloading, and resource allocation decisions (PTORADs). This design enables the algorithm to capture inter-vehicle dependencies more effectively, improves convergence stability, and achieves superior in total computational cost.
- To more accurately capture real-world scenarios, considering the time-varying characteristics of the studied model, parameters like vehicle task size, complexity, maximum execution delay, and available computational power for both ICVs and MECs are defined as interval values. We conducted simulation experiments across multiple parameter settings to demonstrate the efficiency and advancement of the presented strategy.

The rest of this paper is organized as follows: Section 2 introduces the related works. Section 3 presents the system model and problem formulation. In Section 4, we propose an HHDRL-based algorithm to solve the problem. Section 5 shows the simulation results and analysis. Finally, Section 6 concludes the paper and discusses the future work.

## 2. Related Works

In this context, many scholars have studied how to reasonably schedule vehicle offloading tasks in VECNs, and efficiently use MEC computing resources, ICV computing resources, and communication resources to provide high-performance services. Munawar et al. [4] utilized collaboration between RSUs to offload and partition vehicle tasks through MEC, reducing the computational latency. Yin et al. [6] consider task vehicles offloading tasks to RSUs or other resource-sharing vehicles to maximize the benefits of task vehicles, RSUs, and resource-sharing vehicles. Zhou et al. [7] explored the integrated optimization of computational task offloading and resource allocation within dynamic multi-user MEC systems, with the overarching aim of reducing the overall energy consumption of the MEC infrastructure. Xia et al. [8] collaboratively managed and allocated MEC computing power resources based on MEC and vehicle status, and determined vehicle task offloading decisions. Li et al. [9] proposed an on-demand environmental perception and resource allocation strategy in VECNs, achieving delay minimization and resource utilization maximization via a two-stage convex optimization-assisted DRL framework. Wang et al. [10] minimized the energy–delay overhead by constructing the vehicle task offloading and task migration game problem in a mobile edge computing-assisted vehicle network.

The above-mentioned related research problems are often Non-Convex Optimization problems. Exact algorithms and heuristic algorithms, as relatively mature theories, are used by many scholars to solve such problems. For example, Ning et al. [11] used a branch-and-bound algorithm to address the computational offloading quandary for single-user scenarios and employed an iterative heuristic MEC resource allocation algorithm to make the multi-user offloading decision. Liang et al. [12] used a branch-and-bound-based method to obtain the optimal offline solution for tasks under fully predictable assumptions. Chen et al. [13] modeled the vehicle task offloading problem as a constrained generalized allocation model and solved it using the greedy algorithm and the discrete bat algorithm. Liao et al. [14] designed a distributed offloading strategy based on a genetic algorithm (GA) to obtain adaptive offloading decisions and quickly determine the optimal solution for server selection and offloading. Further extensions have explored the integration of traditional optimization techniques with advanced algorithms. For instance, Chen et al. [15] integrated an associative learning immediate memory strategy with genetic operators to develop a multi-objective hybrid GA for task offloading optimization, aiming to minimize average delay, energy consumption, and payment cost. Ref. [16] employed Convolutional Long Short-Term Memory to predict users’ spatio-temporal dynamics and applied dynamic programming to optimize request allocation, thereby ensuring fairness in network edge resource distribution while maintaining service quality. In [17], convolutional neural network-based mobility prediction was combined with a GA to average service latency and maximize edge resource utilization. Moreover, ref. [18] proposed the Energy-Aware job Scheduling at the Edge framework, which leverages model predictive control to manage renewable energy and local computing resources, together with distributed consensus to coordinate service migration.

In the exploration of VECNs, the aforementioned approaches have displayed substantial promise. Nonetheless, a notable challenge persists in attaining proficient transfer learning amidst shifting environmental conditions. Moreover, the dynamic characteristics intrinsic to VECNs introduce considerable complexities, presenting obstacles to precise modeling, prediction, and control. Consequently, previous approaches face limitations in scalability and ability to accurately capture real-world scenarios. Progress in machine learning methodologies, particularly DRL and its sophisticated iterations such as the DDQN, multi-agent deep reinforcement learning, and related algorithms has proficiently tackled the modeling intricacies and computational intricacies inherent in conventional optimization-based methods. These strides provide more accessible avenues to attain asymptotically optimal solutions within time-varying and dynamic environments.

Several works conducted research based on reinforcement learning. Zhu et al. [19] proposed a computing offloading scheme based on MDRL to solve the vehicular computation offloading problem in MEC. Qi et al. [20] proposed an enhanced model-free multi-task deep reinforcement learning algorithm, which allocates parallel tasks to vehicles, edge computing nodes, and cloud central servers with different computing power and resources. Wang et al. [21] adopted a double deep Q-network (DDQN) to address the task offloading challenge in response to real-time changes in network conditions due to vehicle mobility. Lee et al. [22] combined the heuristic algorithm with reinforcement learning, leveraging data on vehicle mobility and parking status obtained from the surrounding environment to make judicious resource allocation. Tian et al. [23] developed a load-balanced deep deterministic policy gradient algorithm that integrates a load-optimization strategy to equalize workload distribution across MEC servers and mitigate the effects of uneven vehicle distributions. Zhao et al. [24] designed an unloading algorithm based on deep reinforcement learning to achieve the best compromise between computing speed and privacy protection during MEC system offloading.

To adapt to various complex environments and target requirements, many scholars have developed different improved DRL algorithm frameworks for different complex scenarios. Yang et al. [25] proposed a two-stage strategy based on DRL, comprising an initial stage for generating discrete actions (task offloading) and a subsequent stage for continuous actions (the transmit power of each vehicle) within the context of offloading and resource allocation. Ma et al. [26] developed a virtual platform based on a deep neural network (DNN), combining Lyapunov optimization with DRL to control the stability of long-term task queues and reduce the computation complexity in the vehicle–cloud-assisted MEC network. Zhang et al. [27] integrated digital twin technology and multi-agent learning into a vehicular edge system to uncover collaborative opportunities among diverse vehicles. They introduced a coordination graph-driven multi-agent deep deterministic policy gradient learning approach to optimize vehicle task offloading costs. Mao et al. [28] proposed an actor–critic-based DRL algorithm that employs a convolutional neural network to derive offloading decisions and integrates greedy subcarrier assignment with an improved water-filling power allocation evaluation strategy to optimize vehicular task offloading and resource allocation. Nie et al. [29] introduced a multi-agent reinforcement learning algorithm as well as a multi-agent federated reinforcement learning algorithm, tailored for employment within centralized and semi-distributed frameworks, respectively. Yuan et al. [30] proposed a two-stage optimization algorithm based on DRL to reduce task processing delay and energy consumption. Zhang et al. [31] proposed a reverse affine maximizer auction mechanism for task allocation and further developed RAMANet, based on a deep learning transformer framework, to fit an exponential number of allocation solutions.

In the aforementioned research endeavors, the dynamic aspects of the environment were not adequately considered. For instance, the mobility patterns of vehicles, the varying characteristics of offloading tasks at different time points, and the available computational power of both vehicles and MEC changed due to the varying tasks being processed at different times.

## 3. System Model and Problem Formulation

**Figure 1:**
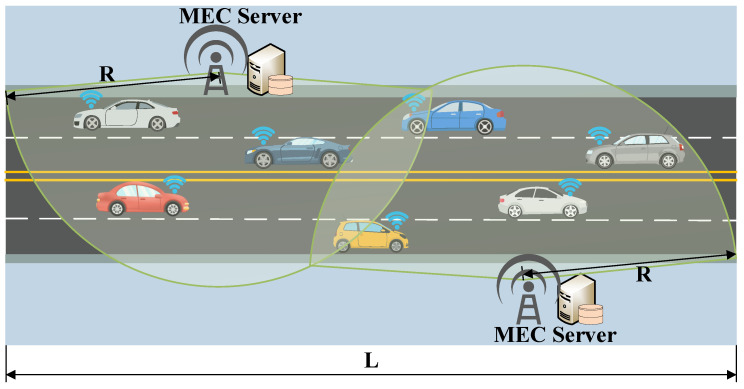
Vehicle–road collaborative edge computing network.

We consider a typical vehicle–road collaborative edge computing network, as shown in Figure 1. Assume that the length of the road is *L*, and the width is *W*. There are *Q* lanes in both directions, and each lane has a width of *H*. *M* RSUs are uniformly distributed on both sides of the road. The coverage radius of each RSU is *R*, and the distance between every two RSUs is 2R. Each RSU is equipped with one MEC server. In practice, the coverage areas of adjacent RSUs may overlap to ensure continuous connectivity for ICVs. For modeling simplicity, we assume that each task generated by an ICV at a given time slot can be offloaded to and executed by only one MEC server. Although each MEC server can serve multiple ICVs simultaneously, a specific task is processed by a single MEC server without partitioning across multiple edges. We denote the set of ICVs and the set of MEC servers in the network as N={1,2,…,N} and M={1,2,…,M}, respectively.

The research considers the whole duration T={1,2,…,T}, which consists of *T* time slots. Besides serving ICVs, MEC servers are also responsible for providing other traffic participants with remote real-time computing and information retrieval services. The available computational capacity of MEC varies over different time slots. Given the brief duration of each time slot, the available computational capability within a specific time slot can be considered constant.

As the ICV transitions from the service area of one RSU to another, a handover occurs. It is imperative to guarantee that the offloading tasks initiated by the ICV are processed and the results transmitted back within the coverage area of a single RSU; otherwise, the task is regarded as unsuccessful. To address this issue, we assume that each ICV completes its task processing within the coverage range of a single RSU. This ensures that the total task execution delay does not exceed the vehicle’s dwell time in the RSU’s service area, thereby avoiding handover interruptions and guaranteeing timely result transmission. Such an assumption is reasonable for short-duration and latency-critical tasks, whose processing time is typically much shorter than the RSU dwell time.

### 3.1. Task Model

At each time slot, each ICV generates a computation task that can be partially executed on the vehicle and partially offloaded to the MEC server. We select three elements that represent the fundamental characteristics of the task and the execution time requirements to represent the task model [32]. The offloading task of the *i*-th ICV at time slot *t* can be expressed as

(1) $$F_{i}\left(t\right)=\left\{s_{i}^{V}\left(t\right),c_{i}^{V}\left(t\right),w_{i}^{V}\left(t\right)\right\},\begin{array}{ccc} & & i\in N, \end{array}$$

where $s_{i}^{V}\left(t\right)$ is the size of the offloading task, $c_{i}^{V}\left(t\right)$ is the computational complexity, and $w_{i}^{V}\left(t\right)$ is the maximum execution delay of the task $F_{i}\left(t\right)$.

### 3.2. Communication Model

When an ICV enters the coverage area of an RSU, it can opt to offload certain tasks to the MEC server via a reliable Vehicle-to-Infrastructure (V2I) communication connection facilitated by the RSU. The study temporarily does not consider the communication occlusion between ICVs and MEC servers in the road environment. We assume that the underlying communication system ensures bandwidth allocation and related functions. The physical heights of the ICVs and MEC servers are disregarded, as we assume they are positioned within a 2D coordinate plane. The coordinates of each MEC server are fixed and defined as $L\left(j\right)={\left[x_{j},y_{j}\right]}^{T},\;\forall j\in M$. Each ICV remains in motion.

At the beginning of the time slot t, the ICVs’ position coordinates are denoted as $V_{i}\left(t\right)={\left[x_{i}\left(t\right),y_{i}\left(t\right)\right]}^{T},\;\forall i\in N$, and at the end of it, they are denoted as $V_{i}\left(t+1\right)={\left[x_{i}\left(t+1\right),y_{i}\left(t+1\right)\right]}^{T},\;\forall i\in N$, which is also the initial position of the vehicle for the next time slot. Similar to [33], the channel gain between the *i*-th ICV and the *j*-th MEC server at time slot *t* can be expressed as

(2) $$g_{ij}\left(t\right)=\alpha_{0}d_{ij}^{-2}\left(t\right)=\frac{\alpha_{0}}{{∥V_{i}\left(t\right)-L_{j}∥}^{2}},$$

where $\alpha_{0}$ denotes the channel gain value per unit reference distance, and $d_{ij}\left(t\right)$ denotes the linear distance between the *i*-th ICV and the *j*-th MEC server at time slot *t*.

The channel gain in this study is dynamically calculated based on the real-time distance between the vehicle’s current position and the MEC server and is reflected in the transmission rate. Since the ICV’s displacement within a single time slot is only a few meters, the resulting variation in gain is negligible; therefore, the channel state information within each slot is determined by the distance at the slot’s start. It is further assumed that channel access, scheduling, and interference management are supported by the underlying communication protocols, allowing the focus to remain on evaluating the HHDRL algorithm’s task offloading and resource allocation performance under such dynamic link conditions.

In line with the *Shannon* theory, the data transfer rate between the *i*-th ICV and the *j*-th MEC server at time slot *t* is given as

(3) $$R_{ij}\left(t\right)=x_{ij}\left(t\right)B\log_{2}\left(1+\frac{p_{ij}\left(t\right)g_{ij}\left(t\right)}{\sigma^{2}+P_{loss}}\right),$$

where $x_{ij}\left(t\right)\in \left\{0,1\right\}$ represents the communication status between the *i*-th ICV and the *j*-th MEC server at time slot *t* when $x_{ij}\left(t\right)=1$ indicates that the *i*-th ICV communicates with the *j*-th MEC server; otherwise $x_{ij}\left(t\right)=0$. *B* denotes the system communication bandwidth. $p_{ij}\in \left[0,\;p_{max}\right]$ denotes the communication transmission power between the *i*-th ICV and the *j*-th MEC at time slot *t*. $\sigma^{2}$ denotes the Gaussian white noise power, and $P_{loss}$ denotes the transmission loss.

### 3.3. Computation Model

For each ICV, the tasks eligible for offloading can either be computed entirely on the VT, termed as local computation, or be partially or completely offloaded to nearby MEC servers, known as offloaded computation. The computational cost for each task encompasses two components: time cost and energy cost.

In this study, each task is modeled as a continuously divisible workload, which can be proportionally executed at both the ICV and the MEC server. This assumption follows the commonly adopted partial offloading paradigm in mobile edge computing, where the total computational load can be split into multiple independent portions for local and edge processing [34,35,36]. Such an abstraction serves as a general representation of parallelizable tasks, including layer-wise deep neural network inference, modular video analytics, and feature-level cooperative perception.

#### 3.3.1. Local Computation

At time slot *t*, the time consumption and energy consumption required by the onboard computing unit to complete the task local computational volume of the *i*-th ICV can be expressed as

(4) $$T_{i}^{loc}\left(t\right)=\frac{\left(1-\theta_{i}\left(t\right)\right)▪s_{i}^{V}\left(t\right)▪c_{i}^{V}\left(t\right)}{f_{i}^{V}\left(t\right)},\;\forall i\in N,$$

(5) $$E_{i}^{loc}\left(t\right)=\kappa ▪\left(1-\theta_{i}\left(t\right)\right)▪s_{i}^{V}\left(t\right)▪c_{i}^{V}\left(t\right)▪{\left(f_{i}^{V}\left(t\right)\right)}^{2},\;\forall i\in N,$$

where $\theta_{i}\in \left[0,\;1\right]$ is the task offloading ratio of the *i*-th vehicle at time slot *t* to the MEC server, $f_{i}^{V}\left(t\right)$ is the local computing capacity of the *i*-th vehicle at time slot *t*, and κ denotes the energy coefficient, which depends on the integrated chip structure.

#### 3.3.2. Offload Computation

The time and energy consumption of the MEC server for completing task offloading is divided into three phases: the task data is transmitted to the MEC (task upload), the task is computed at the MEC (task computational), and the processed results are transmitted back to the MEC server (result return). Since the amount of processed result data transmitted back is small, we ignore the time delay and energy consumption of the return process [37,38].

The task uploading time consumption and energy consumption of the *i*-th vehicle at time slot *t* can be expressed as

(6) $$T_{i}^{tran}\left(t\right)=\frac{s_{i}^{V}\left(t\right)▪\theta_{i}\left(t\right)}{R_{ij}\left(t\right)},$$

(7) $$E_{i}^{tran}\left(t\right)=T_{i}^{tran}\left(t\right)▪p_{ij}\left(t\right).$$

At time slot *t*, the task computational time and energy consumption of the *i*-th ICV in the *j*-th MEC server can be expressed as

(8) $$T_{i}^{MEC}\left(t\right)=\frac{\theta_{i}\left(t\right)▪s_{i}^{V}\left(t\right)▪c_{i}^{V}\left(t\right)}{\beta_{ij}\left(t\right)▪f_{j}^{M}\left(t\right)},$$

(9) $$E_{i}^{MEC}\left(t\right)=T_{i}^{MEC}\left(t\right)▪p_{j}^{M}\left(t\right)▪\beta_{ij}\left(t\right).$$

In Equation (8), $f_{j}^{M}\left(t\right)$ denotes the available computational capability of the *j*-th MEC server. $\beta_{ij}\left(t\right)\in \left[0,\;1\right]$ denotes the percentage of computational capability occupied by the tasks of the *i*-th vehicle in the *j*-th MEC server. $p_{j}^{M}\left(t\right)$ denotes the maximum power of the *j*-th MEC server at time slot *t* in Equation (9).

The total time cost and total energy cost for completing the offloading task of the *i*-th ICV at time slot *t* are shown as

(10) $$T_{i}^{off}\left(t\right)=T_{i}^{tran}\left(t\right)+T_{i}^{MEC}\left(t\right),$$

(11) $$E_{i}^{off}\left(t\right)=E_{i}^{tran}\left(t\right)+E_{i}^{MEC}\left(t\right).$$

#### 3.3.3. Total Computation Cost

To comprehensively evaluate the overall performance of task execution, we define the total computation cost as a weighted combination of the time cost and energy cost. This modeling approach aims to jointly enhance the system’s responsiveness and energy efficiency in VECNs, thereby achieving a coordinated balance between latency and energy consumption.

The time cost represents the end-to-end execution latency of a task from generation to completion. Although each task is constrained by its maximum execution delay, ensuring that all tasks meet their latency limits only guarantees the feasibility of operation rather than global optimality. If only latency constraints are satisfied without optimizing the overall time cost, the system may operate persistently under a critical-load condition, where most tasks are completed just before their maximum execution delay. This leads to increased average latency, MEC server congestion, and low resource utilization efficiency. Minimizing the time cost allows for smoother global task scheduling and improved overall responsiveness and stability. The energy cost characterizes the end-to-end energy consumption during task execution. With the increasing computational complexity of tasks in ICVs, sustained high-power operation results in excessive energy consumption, which not only reduces operational efficiency but also affects the sustainable operation of onboard devices and MEC servers. Incorporating the energy cost into the system design enables energy-efficient operation while maintaining task execution quality, effectively reducing total energy expenditure and improving system energy efficiency and resource utilization.

In summary, jointly considering time cost and energy cost within the total computation cost model provides a more comprehensive characterization of system latency and energy consumption during task execution, serving as a unified quantitative basis for the subsequent modeling of task offloading and resource allocation problems.

At time slot *t*, the time cost $TC_{i}\left(t\right)$ and energy cost $EC_{i}\left(t\right)$ for completing the task of the *i*-th ICV are expressed by Equations (12) and (13), respectively. And the total computation cost is shown as Equation (14).

(12) $$TC_{i}\left(t\right)=\max\left\{T_{i}^{loc}\left(t\right),T_{i}^{off}\left(t\right)\right\},$$

(13) $$EC_{i}\left(t\right)=E_{i}^{loc}\left(t\right)+E_{i}^{off}\left(t\right),$$

(14) $$U_{i}\left(t\right)=\alpha \delta_{1}TC_{i}\left(t\right)+\left(1-\alpha \right)\delta_{2}EC_{i}\left(t\right),$$

where α∈[0, 1] is a weight parameter, which represents the weight of the time cost in the total cost. $\delta_{1}$ and $\delta_{2}$ are the unit costs of delay and energy consumption, respectively.

### 3.4. Problem Formulation

We investigate the optimization problem of task offloading scheduling and resource allocation (TOSRA). Under the constraints of task execution delay and resource limitation, among others, the TOSRA considers the overall cost, including computational time and energy consumption, of the proposed system. The research objective of the TOSRA is to minimize the overall computational cost of tasks for *N* vehicles within a given continuous time, *T*, while ensuring the completion of tasks. Given the above models, we can formulate the TOSRA problem as follows:

(15) $$\begin{array}{cc} \min\; & \sum_{t=1}^{T}\sum_{i=1}^{N}U_{i}\left(t\right)\\ s.t.\; & C1:\;x_{ij}\left(t\right)\in \left\{0,1\right\},\\ \; & C2:\;\sum_{i=1}^{N}\sum_{j=1}^{M}x_{ij}\left(t\right)=1,\\ \; & C3:\;V_{i}\left(t\right)\in \left\{\left(x_{i}\left(t\right),y_{i}\left(t\right)\right)|x_{i}\left(t\right)\in \left[0,L\right],y_{i}\left(t\right)\in \left[0,W\right]\right\},\\ \; & C4:\;0\leq \beta_{ij}\left(t\right)\leq 1,\\ \; & C5:\;T_{i}^{\mathrm{loc}}\left(t\right)\leq w_{i}^{V}\left(t\right),\\ \; & C6:\;T_{i}^{\mathrm{offload}}\left(t\right)\leq w_{i}^{V}\left(t\right),\\ \; & C7:\;∥V_{i}\left(t\right)-L_{j}{∥}^{2}\leq R. \end{array}$$

In Equation (15), constraints C1 and C2 signify that each vehicle can only communicate with at most one MEC server during time slot t, and tasks for each vehicle during time slot t can only be allocated to that specific MEC server. Constraint C3 ensures that the position of each vehicle remains within the specific road range. Constraint C4 denotes that the resource utilization of each MEC server does not exceed 1. Constraints C5 and C6 enforce that the local computation time and offloading computation time for tasks during time slot t do not surpass the maximum execution delay. According to constraint C7, the Euclidean distance between vehicles and MEC servers should not be greater than the service radius of the MEC server.

Equation (15) represents an optimization problem that takes into account both time cost and energy costs. It is worth noting that dynamically obtaining an optimal solution for such a time-varying problem is computationally intractable. Therefore, instead of using conventional optimization methods, we utilize deep reinforcement learning methods to solve the problem, which will be illustrated in the next section.

## 4. HHDRL-Based Task Offloading and Resource Allocation

**Figure 2:**
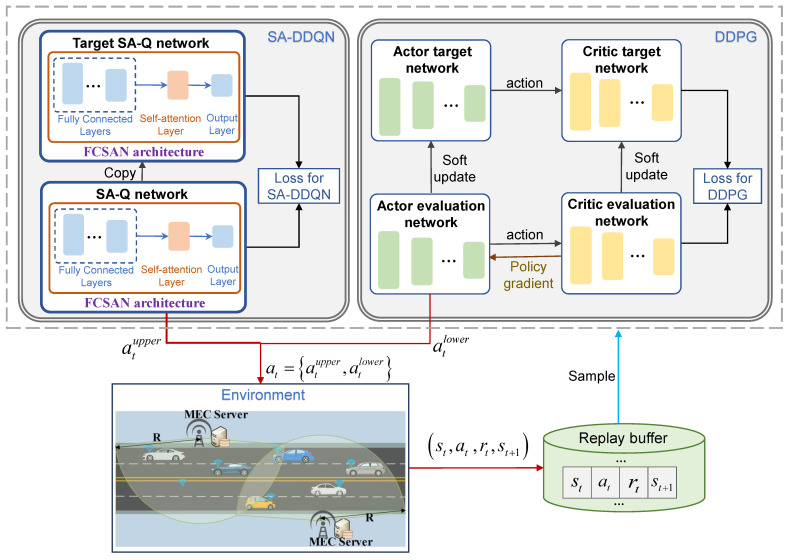
HHDRL algorithm framework.

As described in Section 3, the TOSRA problem involves both discrete and continuous control variables. Directly solving this mixed action space using a unified DRL framework would increase learning complexity and reduce convergence efficiency. To address this challenge, we propose a hybrid hierarchical deep reinforcement learning (HHDRL) algorithm. In HHDRL, the upper layer employs a self-attention-enhanced DDQN (SA-DDQN) to make discrete control variables, while the lower layer uses a DDPG algorithm to handle continuous control variables. This hierarchical decomposition improves learning efficiency and aligns with practical execution logic, where communication access must be determined before offloading and resource scheduling. Figure 2 illustrates the overall framework of HHDRL.

### 4.1. Problem Analysis

Assume that within the duration T, the length of each time slot is Δt. In each time slot, each ICV updates its location, generates a new task request, and updates the local available computing power. Additionally, each MEC server also updates its current available computational power. The agent makes effective action decisions based on the observed system states, including the task offloading decision $\left\{x_{ij}\left(t\right)\right\}$ (ICV chooses which MEC to offload the task to), task offloading ratio $\left\{\theta_{i}\left(t\right)\right\}$ (percentage of ICV task offloading), communication power $\left\{p_{ij}\left(t\right)\right\}$ (communication power between ICV and corresponding MEC), and MEC computational power allocation ratio $\left\{\beta_{ij}\left(t\right)\right\}$ (percentage of available elastic computational power allocated to the communicating ICV).

In the action decision tuple, $\left\{x_{ij}\left(t\right)\right\}$ is a discrete control variable, and $\left\{\theta_{i}\left(t\right)\right\}$, $\left\{p_{ij}\left(t\right)\right\}$, and $\left\{\beta_{ij}\left(t\right)\right\}$ are continuous control variables. To address the challenge of handling both discrete and continuous control variables, we decompose the entire optimization problem into two sub-processes that optimize decisions alternately. The upper layer involves the communication decision (CD) process that generates discrete control variables, while the lower layer involves the power control, task offloading, and resource allocation decision (PTORAD) process that generates continuous control variables.

- **Upper layer—CD process**: The upper layer agent is utilized to learn how to make optimal communication decisions. The agent observes information such as vehicle distance states, vehicle task status, vehicle and nearby MEC servers’ computing power status, etc. It employs reinforcement learning algorithms to determine offloading decision actions $\left\{x_{ij}\left(t\right)\right\}$. The task offloading decision of each vehicle will impact the resource allocation decisions in the lower layer.
- **Lower layer—PTORAD process**: The upper-layer actions act on the environment. Based on the observed changes in system state, the lower layer agent adjusts its actions: $\left\{\theta_{i}\left(t\right)\right\}$, $\left\{p_{ij}\left(t\right)\right\}$, and $\left\{\beta_{ij}\left(t\right)\right\}$. The adjustment aims to enhance the overall reward performance of the actions in both layers. If the offloading decision corresponding to the *i*-th vehicle is $x_{ij}\left(t\right)=0,$ the lower-layer policy treats the corresponding action as an invalid action, set to 0. If $x_{ij}\left(t\right)=1$, the lower layer generates valid actions.

### 4.2. HHDRL Framework

As discussed above, we propose the HHDRL algorithm to effectively handle the coexistence of discrete and continuous variables. As shown in Figure 2, the HHDRL is a two-layer algorithm: (1) the upper layer derives the CDs by exploiting the SA-DDQN algorithm and (2) the lower layer determines the PTORADs by utilizing the DDPG algorithm at the same time. The following is a detailed description of the HHDRL framework.

#### 4.2.1. States, Actions, and Rewards

Like DRL, the HHDRL algorithm also consists of three key elements: state space S, action space A, and reward R. During each step *t*, the agent chooses an action $a_{t}$ from the action space A under the current state $s_{t}$, executes the action $a_{t}$, and receives a reward $r_{t}$. Then the current state $s_{t}$ moves to the next state $s_{t+1}$. The key elements of the HHDRL algorithm are discussed below.

**State space**: At each time interval, the vehicle position, task size, complexity, maximum execution delay, and available computational capacity for both ICVs and MECs are changing. Therefore, the state space, denoted as S, comprises these elements. At time slot *t*, the state $s_{t}\in S$ is defined as

(16) $$s_{t}=\left\{\left\{V_{i}\left(t\right)\right\},\left\{s_{i}^{V}\left(t\right)\right\},\left\{c_{i}^{V}\left(t\right)\right\},\left\{w_{i}^{V}\left(t\right)\right\},\left\{f_{i}^{V}\left(t\right)\right\},\left\{f_{j}^{M}\left(t\right)\right\}\right\},$$

where $V_{i}\left(t\right)$ represents the vehicle’s location information. We map the change in the vehicle’s position to the distance between the vehicle and RSU. $s_{i}^{V}\left(t\right)$, $c_{i}^{V}\left(t\right)$, and $w_{i}^{V}\left(t\right)$ represent the task size, computational complexity, and the maximum execution delay of the *i*-th vehicle at time slot *t*, respectively. $f_{i}^{V}\left(t\right)$ represents the available computational capacity of the *i*-th vehicle at time slot *t*, and $f_{j}^{M}\left(t\right)$ represents the available computational capacity of the *j*-th MEC at time slot *t*.

**Action space**: The upper layer of HHDRL generates the action $a_{t}^{upper}=\left\{x_{ij}\left(t\right)\right\}$, and the lower layer generates the action $a_{t}^{lower}=\left\{\left\{\theta_{i}\left(t\right)\right\},\left\{p_{ij}\left(t\right)\right\},\left\{\beta_{ij}\left(t\right)\right\}\right\}$. Both actions collectively influence the environmental system. Therefore, the action $a_{t}\in A$ is referred to as the overall action, and it is given by

(17) $$a_{t}=\left\{a_{t}^{\mathrm{upper}},a_{t}^{\mathrm{lower}}\right\}=\left\{\left\{x_{ij}\left(t\right)\right\},\left\{\theta_{i}\left(t\right)\right\},\left\{p_{ij}\left(t\right)\right\},\left\{\beta_{ij}\left(t\right)\right\}\right\}.$$

**Reward function**: HHDRL aims to obtain and maximize the system’s long-term cumulative discounted reward. The reward function follows a violation-means-failure design principle: only when all constraints (C1–C7) are satisfied is a negative reward constructed based on the total computation cost $U_{i}\left(t\right)$ to drive the minimization of the total cost. Once any constraint is violated, a unified large negative reward −*X* is directly assigned. This design reflects the causal aggregation of task execution, as any improper control of actions, as well as communication coverage failure, ultimately results in tasks not being completed on time. Therefore, adopting a unified “failure penalty” mechanism effectively captures different sources of constraint violations while enhancing training stability and interpretability. Thus, at time *t*, the reward $r_{t}$ is expressed as

(18) $$r_{t}=\left\{\begin{array}{cc} -\sum_{i=1}^{N}U_{i}\left(t\right), & \mathrm{if}\;C1\;\mathrm{to}\;C7\;\mathrm{are}\;\mathrm{satisfied},\\ -X, & \mathrm{otherwise}. \end{array}\right.$$

The cumulative discounted reward is the sum of all rewards from the current moment to the end of a training episode. At time slot *t*, the cumulative discounted reward of the system is expressed as

(19) $$R_{t}=r_{t}+\gamma r_{t+1}+\gamma^{2}r_{t+2}+\ldots ,$$

where γ∈[0,1] is the discount factor.

Given the above discussion, the agent selects better actions to obtain greater rewards through learning a policy function π. We utilize the action-value function $Q_{\pi }\left(s_{t},a_{t}\right)$ to evaluate and improve the policy π, which is also referred to as the *Q*-function. The $Q_{\pi }\left(s_{t},a_{t}\right)$ is formulated as

(20) $$Q_{\pi }\left(s_{t},a_{t}\right)=E\left[R_{t}|s_{t},a_{t}\right]=E\left[r_{t}+\gamma Q_{\pi }\left(s_{t+1},a_{t+1}\right)|s_{t},a_{t}\right].$$

And the *Q*-value is the value estimated for specific state–action pairs in the *Q*-function. Under the optimal policy $\pi^{*}$, the *Q*-function has the highest *Q*-value.

#### 4.2.2. SA-DDQN for Discrete Action in Upper Layer

Building upon the preceding discussion, we employ SA-DDQN to obtain the discrete variable: task offloading decisions $\left\{x_{ij}\left(t\right)\right\}$. SA-DDQN is an improvement on the traditional DDQN algorithm, which is enhanced by a self-attention mechanism. This refinement facilitates the attainment of optimal communication decisions.

SA-DDQN employs a fully connected self-attention network (FCSAN) to approximate the Q-function, integrating fully connected layers with self-attention mechanisms. The FCSAN architecture shown in Figure 2 illustrates its structural composition, which consists of fully connected layers and a self-attention layer. After traversing the initial and subsequent fully connected layers, the input state information generates feature mapping. These mappings reflect the importance of individual features, collectively forming a sequence denoted as $X=\left[x_{1},x_{2},\ldots ,x_{n}\right]$. The self-attention layer exploits the interdependencies among input features to autonomously determine the weights assigned to each feature. In this study, with a considerable amount of input state information, the utilization of the self-attention layer enables the neural network to focus more on crucial and informative features. This enhancement leads to improved output quality and reduces redundant information stemming from multiple features.

The calculation principle of the self-attention layer is as follows: Let $X=[x_{1},x_{2},\ldots ,x_{n}]$ denote the input for the self-attention layer. The *Q* (Query), *K* (Key), and *V* (Value) matrices are derived by applying the linear transformation matrices $W_{q}$, $W_{k}$, and $W_{v}$, as follows:

(21) $$\begin{array}{cc} \; & Q=W_{q}X,\\ \; & K=W_{k}X,\\ \; & V=W_{v}X, \end{array}$$

where $W_{q}$, $W_{k}$, and $W_{v}$ represent the parameter matrices that need to be learned.

We calculate the dot products of the matrix *Q* and $K^{T}$. In order to prevent the dot product from being too large, we divide it by $\sqrt{d_{k}}$. Subsequently, we normalize using the softmax function, calculating the correlation coefficients between various features of the current input. Finally, we perform an element-wise multiplication of matrix *V* with its corresponding attention weights, obtaining the final output *Z* [39]. As shown in Equation (22),

(22) $$Z=\mathrm{softmax}\left(\frac{QK^{T}}{\sqrt{d_{k}}}\right)V,$$

where $d_{k}$ represents the vector dimensionality of matrices *Q* and *K*.

SA-DDQN has an SA-Q network and a target SA-Q network, which serve as the evaluation network and the target network, respectively. They both adopt the FCSAN structure. The two networks are used to select the action corresponding to the maximum *Q*-value and to evaluate the *Q*-value of the best action, respectively. This decouples the action selection from the policy evaluation. The generated *Q*-values from the SA-Q network and target SA-Q network are denoted as $Q(s_{t},a_{t}^{upper};\eta )$ and $Q(s_{t},a_{t}^{upper};\eta^{\prime })$, where η is the SA-Q network parameters and $\eta^{\prime }$ is the target SA-Q parameters.

The agent in SA-DDQN utilizes the ε-greedy policy to determine the optimal action $a_{t}^{upper*}$ for the state $s_{t}$, which corresponds to the action with the maximum *Q*-value from the SA-Q network. The $a_{t}^{upper*}$ is formulated as follows:

(23) $$a_{t}^{upper*}=\mathrm{argma}x_{a_{t}^{upper}}Q\left(s_{t},a_{t}^{upper};\eta \right).$$

Then, the agent exploits the target SA-Q network to calculate the *Q*-value using the action $a_{t}^{upper*}$, which is written as

(24) $$y_{t}^{SA-DDQN}\;=\;r_{t}\;+\;\gamma Q\left(s_{t+1},\underset{a_{t}^{upper}}{\mathrm{argmax}}\;Q\left(s_{t+1},a_{t+1}^{upper};\;\eta \right);\;\eta^{\prime }\right).$$

Thus, the SA-DDQN is trained through minimizing the loss function shown as follows:

(25) $$L\left(\eta \right)={\left(y_{t}^{SA-DDQN}-Q\left(s_{t},a_{t}^{upper};\eta \right)\right)}^{2}.$$

In initialization, the SA-Q network and the SA-Q target network share identical structures and parameter configurations. Throughout the training process, the parameters of the SA-Q network undergo continuous optimization, with updates periodically transferred to the SA-Q target network at specified intervals.

#### 4.2.3. DDPG for Continuous Actions in Lower Layer

In order to address the control problems of continuous actions—$\left\{\theta_{i}\left(t\right)\right\}$, $\left\{p_{ij}\left(t\right)\right\}$, and $\left\{\beta_{ij}\left(t\right)\right\}$—there are usually two approaches. One of the approaches involves discretizing continuous control and then using the previously mentioned DQN or DDQN method for training. However, as control precision increases, the number of discrete actions grows exponentially, leading to the curse of dimensionality. A better approach is to directly use the continuous control methods. DDPG is a DRL algorithm based on policy gradients and is a commonly used method for solving continuous control problems.

DDPG adopts an actor–critic architecture, in which two DNNs are established, serving as the actor network μ (policy network) and the critic network *q* (value network) separately, to divide the exploration and learning updates of the action policy network. The actor network controls the movements of the agent, generating an action based on the state $s_{t}$. Within the actor network, there are two networks: the actor evaluation network $\mu (s_{t};\omega )$ and the actor target network $\mu (s_{t};\omega^{\prime })$, where ω is the actor evaluation network parameters and $\omega^{\prime }$ is the actor target network parameters. On the other hand, the critic network does not directly control the agent but rather scores actions for the given state $s_{t}$, guiding the policy network for improvements. The critic network also consists of two networks, which are the critic evaluation network $q(s_{t},a_{t}^{lower};\varphi )$ and the critic target network $q(s_{t},a_{t}^{lower};\varphi^{\prime })$, where φ is the critic evaluation network parameters and $\varphi^{\prime }$ is the critic target network parameters.

The training objective of DDPG is to maximize the expected *Q*-value of the actor network, denoted as J(ω), and minimize the expected loss function of the critic network, denoted as L(φ), as shown in the following equations:

(26) $$\left\{\begin{array}{cc} \max_{\omega }\;J\left(\omega \right)\; & =\;\max_{\omega }E\;\left[q(s_{t},a_{t};\varphi )\right]\;=\;\max_{\omega }E\;\left[q(s_{t},\mu \left(s_{t};\omega \right);\varphi )\right]\\ \min_{\theta }J\left(\varphi \right) & =\min_{\theta }E\left[L(\varphi )\right]\\ L(\varphi ) & =\frac{1}{2}{\left(y_{t}^{\mathrm{DDPG}}-q\left(s_{t},a_{t}^{\mathrm{lower}};\varphi \right)\right)}^{2}\\ y_{t}^{\mathrm{DDPG}} & =r_{t}+\gamma q\left(s_{t+1},\mu \left(s_{t+1};\omega^{\prime }\right);\varphi^{\prime }\right) \end{array}\right..$$

The parameters ω and φ are updated using the gradient descent method according to the following equation:

(27) $$\nabla_{\omega }J\left(\omega \right)=\nabla_{\omega }E\left[q(s_{t},\mu \left(s_{t};\omega \right);\varphi )\right]=\frac{1}{\Theta }\sum_{i=1}^{\Theta }\left[\nabla \mu \left(s_{t};\omega \right)\cdot \nabla q\left(s_{t},\mu \left(s_{t};\omega \right);\varphi \right)\right]\left|s_{t}=s_{i}\right.,$$

(28) $$\begin{array}{cc} \nabla_{\varphi }J\left(\varphi \right) & =\nabla_{\varphi }E\left[\frac{1}{2}{\left(y_{t}^{\mathrm{DDPG}}-q\left(s_{t},a_{t}^{\mathrm{lower}};\varphi \right)\right)}^{2}\right]\\ \; & =\frac{1}{\Theta }\sum_{i=1}^{\Theta }\left[\left(r_{t}+\gamma \cdot q\left(s_{t+1},\mu \left(s_{t+1};\omega^{\prime }\right);\varphi^{\prime }\right)\right)\right.\left.-\nabla \mu \left(s_{t};\omega \right)\cdot \nabla q\left(s_{t},\mu \left(s_{t};\omega \right);\varphi \right)\right]\left|s_{t}=s_{i}\right.. \end{array}$$

where Θ is the size of the mini-batch.

For the purpose of ensuring the stability of neural network training, the parameters of the target network of the DDPG algorithm are updated using soft target update to copy the parameters of the actor evaluation network and the critic evaluation network to the actor target network and the critic target network at the end of each round.

#### 4.2.4. Training and Implementation of HHDRL Algorithm

Algorithm 1 shows the comprehensive steps for implementing the HHDRL algorithm. The process initiates with the initialization of network parameters for both the SA-DDQN and DDPG (lines 1–3). Subsequently, the experience replay buffer Φ is initialized (line 4). The training process of the HHDRL encompasses a maximum of $EP_{\max}$ episodes, with each episode comprising *T* steps. Prior to the commencement of each episode, the system’s environment is initialized, yielding the initial environmental state $s_{0}$ (line 6). Within each step of an episode, the CD action $a_{t}^{upper}=\left\{\left\{x_{ij}\left(t\right)\right\}\right\}$ is generated by the SA-DDQN (line 9), while the PTORAD action $a_{t}^{lower}=\left\{\left\{\theta_{i}\left(t\right)\right\},\left\{p_{ij}\left(t\right)\right\},\left\{\beta_{ij}\left(t\right)\right\}\right\}$ is produced by the DDPG (line 11). In instances where $x_{ij}\left(t\right)=0$, the corresponding indices $\theta_{i}\left(t\right),p_{ij}\left(t\right),\beta_{ij}\left(t\right)$ within $a_{t}^{lower}$ are adjusted to 0. Conversely, if $x_{ij}\left(t\right)=1$, the values within $a_{t}^{lower}$ are maintained as is (lines 10–13). The actions $a_{t}^{upper}$ and $a_{t}^{lower}$ are concatenated to form the composite action $a_{t}$ (line 15). Upon execution of $a_{t}$ by the environment, a reward $r_{t}$ is received, and the state transitions to $s_{t+1}$ (line 16). The tuples $\left(s_{t},a_{t},r_{t},s_{t+1}\right)$ are subsequently stored in Φ. It is stipulated that older data groups may be expunged from the experience replay buffer upon it reaching its storage capacity (line 17). A mini-batch of D samples is drawn from Φ for the purpose of training the network parameters of HHDRL (line 18). The subsequent phase involves the training of the SA-DDQN network parameters, η and $\eta^{\prime }$, and the DDPG network parameters, ω, $\omega^{\prime }$, φ, and $\varphi^{\prime }$ (lines 19–23). As the training progresses, each neural network accumulates sufficient experience to optimize and update its parameters, thereby achieving improved global network performance.
**Algorithm 1:** Training process of HHDRL1:Initialize evaluation network parameter η and target network parameter $\eta^{\prime }$ of SA-DDQN;2:Initialize actor evaluation network parameter ω and critic evaluation network parameter φ of DDPG;3:Initialize actor target network parameter $\omega^{\prime }$ and critic target network parameter $\varphi^{\prime }$ of DDPG;4:Initialize the experience replay buffer Φ;5:**for** episode = 1 to $EP_{\max}$ **do**6:  Initialize environment, observe the initial system state $s_{0}$;7:  **for** t=1 to *T* **do**8:    Get the current state $s_{t}$;9:    Selecting upper communication decision actions $a_{t}^{upper}=\left\{\left\{x_{ij}\left(t\right)\right\}\right\}$ based on SA-DDQN;10:    **if** $x_{ij}\left(t\right)=1$ **then**11:     Selecting lower actions task offloading ratio $\theta_{i}\left(t\right)$, communication power $p_{ij}\left(t\right)$, MEC computational power allocation ratio $\beta_{ij}\left(t\right)$ based on DDPG, and $a_{t}^{lower}=\left\{\left\{\theta_{i}\left(t\right)\right\},\left\{p_{ij}\left(t\right)\right\},\left\{\beta_{ij}\left(t\right)\right\}\right\}$;12:    **else if**
$x_{ij}\left(t\right)=0$
**then**13:     Task offloading ratio $\theta_{i}\left(t\right)=0$, communication power $p_{ij}\left(t\right)=0$, MEC computational power allocation ratio $\beta_{ij}\left(t\right)=0$, and $a_{t}^{lower}=\left\{\left\{\theta_{i}\left(t\right)\right\},\left\{p_{ij}\left(t\right)\right\},\left\{\beta_{ij}\left(t\right)\right\}\right\}$;14:    **end if**15:    Configure $a_{t}=\left\{a_{t}^{upper},a_{t}^{lower}\right\}$;16:    Execute action $a_{t}$, obtain reward $r_{t}$, and the next state $s_{t+1}$;17:    Store $\left(s_{t},a_{t},r_{t},s_{t+1}\right)$ into Φ, and remove the oldest tuple when the buffer reaches capacity;18:    Sample a mini-batch of D samples from Φ;19:    Obtain *Q*-value of SA-DDQN according to Equation (24);20:    Update η by Equation (25); and reset $\eta^{\prime }\leftarrow \eta$ periodically;21:    Obtain *Q*-value of DDPG and update minimizing the expected loss function of the critic network according to Equation (26);22:    Update actor evaluation network parameter ω and critic evaluation network parameter φ according to Equation (27) and Equation (28) respectively;23:    Update parameter $\omega^{\prime }$ and $\varphi^{\prime }$ by using soft target update;24:   **end for**25:**end for**

**Figure 3:**
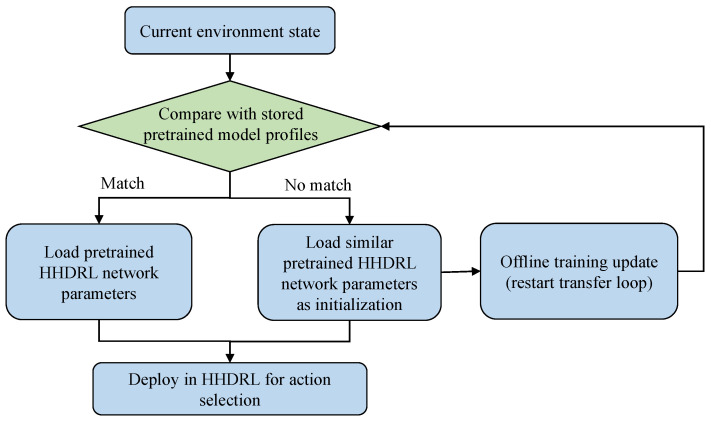
Workflow of the pretrained model transfer strategy in HHDRL.

In the implementation of the HHDRL algorithm, agents select actions based on the observed state of the environment by leveraging a pretrained network rather than starting from random initialization. Figure 3 shows the Workflow of the pretrained model transfer strategy in HHDRL. Specifically, the parameters of the SA-DDQN and DDPG modules are initialized using previously trained models under comparable system settings, such as similar vehicular densities and MEC resource levels. When a pretrained model that closely matches the current environment is available, its parameters are directly adopted without further adaptation. If no perfectly matched model exists, a model trained under similar settings is used as the initialization point, followed by limited fine-tuning to enable fast convergence. This hierarchical reuse strategy not only accelerates convergence and mitigates the instability of early-stage exploration but also ensures compatibility of model structure and parameters. In practice, the decision of whether to reuse a model directly or fine-tune it is based on the similarity between the current environment statistics and the stored profiles of past environments, ensuring that only models with close relevance are transferred. This approach improves reproducibility by explicitly specifying how pretrained models are selected and adapted. Consequently, the HHDRL algorithm achieves low application latency, which is particularly advantageous in vehicular scenarios. Moreover, the algorithm is trained offline, with model reuse or adaptation performed intermittently when significant environmental changes occur.

### 4.3. Complexity and Scalability Analysis

#### 4.3.1. Time Complexity Analysis

The time complexity of the HHDRL algorithm mainly arises from the upper-layer SA-DDQN and the lower-layer DDPG. In the SA-DDQN, the input state is first processed by several fully connected layers for feature extraction, followed by a self-attention layer to model the correlations between ICVs and MECs. Let the input dimension be $d_{\mathrm{in}}$, the number of hidden units be *h*, the number of fully connected layers be $L_{\mathrm{fc}}$, the output action dimension be *a*, and the length of the attention input sequence be *k* (approximately N+M in the worst case). The complexity of the fully connected layers is $O\left(d_{\mathrm{in}}h+\left(L_{\mathrm{fc}}-1\right)h^{2}+ha\right)$, while the self-attention layer has a complexity of $O(kh^{2}+k^{2}h)$. Therefore, the overall time complexity of SA-DDQN is given by

(29) $$O(d_{\mathrm{in}}h+\left(L_{\mathrm{fc}}-1\right)h^{2}+ha+kh^{2}+k^{2}h),$$

where k=O(N+M) in the worst case, and the complexity increases linearly to quadratically with the number of ICVs and MECs.

DDPG employs an actor network to generate continuous actions and a critic network to evaluate state–action pairs. Let the input state dimension be $d_{\mathrm{in}}$, the action dimension be $a_{c}$, the number of actor layers be $L_{a}$, and the number of hidden units in the actor be $h_{a}$. Similarly, let the number of critic layers be $L_{c}$, and the number of hidden units in the critic be $h_{c}$. The complexity of the actor network is approximately $O(d_{\mathrm{in}}h_{a}+\left(L_{a}-1\right)h_{a}^{2}+h_{a}a_{c})$, while the critic network has a complexity of approximately $O(\left(d_{\mathrm{in}}+a_{c}\right)h_{c}+\left(L_{c}-1\right)h_{c}^{2}+h_{c})$. Thus, the overall time complexity of the DDPG is as follows:

(30) $$O(d_{\mathrm{in}}h_{a}+\left(L_{a}-1\right)h_{a}^{2}+h_{a}a_{c}+\left(d_{\mathrm{in}}+a_{c}\right)h_{c}+\left(L_{c}-1\right)h_{c}^{2}+h_{c}).$$

In summary, the overall time complexity of the HHDRL algorithm is the sum of the two components:

(31) $$O(d_{\mathrm{in}}\left(h+h_{a}+h_{c}\right)+ah+a_{c}\left(h_{a}+h_{c}\right)+\left(L_{\mathrm{fc}}-1\right)h^{2}\;+\left(L_{a}-1\right)h_{a}^{2}+\left(L_{c}-1\right)h_{c}^{2}+kh^{2}+k^{2}h+h_{c}),$$

Since the inference stage only involves forward propagation, its execution cost is significantly lower than that of the training stage. Therefore, the proposed algorithm is capable of meeting the real-time requirements of vehicular platforms.

#### 4.3.2. Space Complexity Analysis

In the HHDRL algorithm, the space complexity is mainly determined by the size of the network parameters and the memory required for intermediate tensors during execution. For SA-DDQN, the parameter scale of the fully connected layers is approximately $O(d_{\mathrm{in}}h+\left(L_{\mathrm{fc}}-1\right)h^{2}+ha)$. In the self-attention layer, the projection matrices $W_{q},W_{k},W_{v}$ (each of size h×h) have about $O(3h^{2})$ parameters. During execution, additional memory is required to store *Q*, *K*, and *V* (k×h) and the attention matrix (k×k), resulting in an extra cost of $O(kh+k^{2})$. For DDPG, the parameter scale of the actor network is approximately $O(d_{\mathrm{in}}h_{a}+\left(L_{a}-1\right)h_{a}^{2}+h_{a}a_{c})$, while that of the critic network is $O(\left(d_{\mathrm{in}}+a_{c}\right)h_{c}+\left(L_{c}-1\right)h_{c}^{2}+h_{c})$.

Therefore, the overall space complexity of HHDRL can be expressed as

(32) $$O(d_{\mathrm{in}}h+\left(L_{\mathrm{fc}}-1\right)h^{2}+ha+3h^{2}+d_{\mathrm{in}}h_{a}+\left(L_{a}-1\right)h_{a}^{2}\;+h_{a}a_{c}+\left(d_{\mathrm{in}}+a_{c}\right)h_{c}+\left(L_{c}-1\right)h_{c}^{2}+h_{c}),$$

Since inference only involves storing activations without backpropagation variables, the runtime memory is mainly determined by the network parameter scale, which can fully meet the storage requirements of vehicular embedded platforms.

#### 4.3.3. Scalability Analysis

The HHDRL framework demonstrates good scalability in large-scale scenarios. As the number of vehicles increases, the upper-layer SA-DDQN makes discrete decisions based on system states, while the lower-layer DDPG performs continuous control accordingly. As a result, the overall complexity does not grow exponentially with the global scale but mainly depends on the number of relevant nodes, thereby avoiding excessive computational overhead. When the number of MEC servers increases, the continuous actions of the lower-layer DDPG are performed only for the nodes selected by the upper layer, whereas unselected nodes are not involved in task offloading and resource allocation. This design effectively controls the action dimension and reduces redundant computation. Overall, the hierarchical structure and attention mechanism ensure that HHDRL maintains stable convergence and performance as the numbers of ICVs and MEC servers increase. In terms of deployment, the inference time per step grows approximately linearly with the number of candidate nodes rather than with the overall system scale. Under typical system configurations, as long as the candidate set remains within a reasonable range, real-time requirements can be satisfied, thereby making the framework feasible for deployment in scenarios with dozens of vehicles and a small number of MEC servers.

## 5. Simulation Results and Analysis

In this section, we conduct numerical simulations to evaluate the performance of our proposed HHDRL algorithm and analyze its performance compared with six other benchmark algorithms.

### 5.1. Setting and Benchmarks

For this simulation, we assume that it is conducted on a two-way four-lane highway with a length of 1800m and a width of 15m, as depicted in Figure 1. We use the southwestern corner of Figure 1 as the origin point, with the length of the road as the *x*-axis and its width as the *y*-axis. Each lane has a width of 3m. The ordinate coordinates of the lane centerlines are situated at 2.25, 5.75, 9.25, and 12.75. Scattered along both sides of the road, two RSUs equipped with MEC servers are strategically positioned, and their coordinates are (500, 15) and (1300, 0). The service radius of each RSU is set to 500 m, which aligns with the typical coverage range of existing V2I communication technologies such as LTE-V2X, generally providing reliable connectivity within 300–500 m under typical V2I communication conditions [40]. Beyond this distance, path loss increases approximately with the square of the transmission range, resulting in a rapid degradation of signal quality and increased risks of packet loss and latency jitter. Therefore, a 500 m coverage radius represents a commonly used configuration, and has also been adopted in several recent studies [41,42].

The traffic scenario assumes that 4–12 ICVs travel at a constant speed along a straight multi-lane road, corresponding to a small-to-medium-scale computing environment typically observed in highway or urban arterial settings. This assumption ensures a stable communication topology, eliminates interference caused by vehicle acceleration or lane-changing behaviors, and captures the computational and communication competition among vehicles. It enables the study to focus on optimizing task offloading and resource allocation mechanisms, and has been widely adopted in recent vehicular edge computing research [43,44,45].

A summary of the simulation parameters is presented in Table 1. In the real-world environment, the task attributes generated by each ICV at different times vary, including their size, complexity, and maximum execution delay. Simultaneously, the available computational resources also vary due to the varying computational workload handled by ICVs and MEC servers at different points in time. Therefore, in order to conduct a more authentic simulation of task attributes and computational capabilities for ICVs and MEC servers, this research is different from existing related papers by opting to specify parameters with values within ranges rather than using fixed parameters. Specifically, the task size $s_{i}^{V}\left(t\right)$ is set to 0.5–11 Mbits, based on the 0.1–0.6 Mbits in [34] and 10–90 Mbits in [43]; the task computational complexity is chosen as 1–4.5 Gcycles/Mbit, by referring to the lightweight workload (500–1500 cycles/bit) and heavyweight workload (5000 cycles/bit) in [46]; and the maximum execution delay is 0.5–3 s, guided by the 3 s tolerance in [7] and the 0.8–4 s range in [46]. The selected parameter ranges comprehensively cover the diversity of vehicular computing workloads in real-world ICV applications. Specifically, the task size range (0.5–11 Mbits) represents the data volumes of typical perception and feature-upload tasks. The computational complexity range (1–4.5 Gcycles/Mbit) captures both lightweight feature extraction and heavy deep neural network inference workloads commonly executed on vehicle or MEC processors. Meanwhile, the maximum execution delay range (0.5–3 s) corresponds to real-time perception, decision, and control tasks in safety-critical vehicular environments. Overall, these parameter settings strike a balance between practicality and representativeness, ensuring that the simulated tasks reflect realistic vehicular edge computing demands while maintaining computational tractability for algorithmic performance evaluation. In the simulation experiments, all results related to the total computation cost are obtained by averaging multiple independent runs. Moreover, the incorporation of randomness in task size, task complexity, maximum execution delay constraint, and computing resources ensures that the results are both representative and robust.

The hyperparameter configuration of the HHDRL algorithm is as follows: For the upper-layer SA-DDQN, the first and second fully connected hidden layers of the FCSAN contain 128 and 64 neurons, respectively. The output dimension of the self-attention layer is consistent with that of the second hidden layer (64 dimensions). After self-attention feature fusion, the output layer generates action values, with the number of neurons equal to the action dimension. Each fully connected layer uses the ReLU activation function, and the network parameters are optimized using the Adam optimizer. The target network is updated every 40 iterations. For the lower-layer DDPG, both the actor and critic networks consist of three fully connected hidden layers with 512, 256, and 128 neurons, respectively. The ReLU activation function and Adam optimizer are used for training. The output layer of the actor network adopts the tanh activation function to generate continuous action values, and the target networks are synchronized through a soft update mechanism with an update rate of 0.01.

The proposed HHDRL algorithm is compared with six benchmark algorithms, which are as follows:

**DDQN+DDPG**: Following [34], ICVs utilize a double deep Q-network (DDQN) to determine CD decisions, while DDPG controls the generation of PTORADs, similar to the lower-level algorithm in HHDRL.

**DQN+DDPG**: Following [25], ICVs utilize a deep Q-network (DQN) to determine CDs, while DDPG controls the generation of PTORADs, similar to the lower-level algorithm in HHDRL.

**IGA**: Based on [14], an Improved Genetic Algorithm (IGA) is adopted, in which the standard GA is adapted to address the joint task offloading and resource allocation problem considered in this work.

**Random+DDPG**: Referencing [25], ICVs randomly decide whether to offload tasks to nearby MEC servers and employ DDPG to determine the strategy for PTORADs for successfully offloaded tasks, similar to the lower-level algorithm in HHDRL.

**Full Offloading**: Referencing [34], ICVs offload all tasks to nearby MEC servers for computation. Tasks that cannot be successfully offloaded due to distance are computed locally within the vehicles. DDPG is used to generate PTORADs for successfully offloaded tasks.

**Local Computation**: Referencing [25,34], all ICVs compute their tasks locally within the vehicles, without offloading to any MEC servers.

**Random Offloading and Resource Allocation (RORA)**: Referencing [43], ICVs make random decisions regarding task offloading, as well as PTORADs, under environmental constraints.

### 5.2. The Performance of the Proposed Algorithm

**Figure 4:**
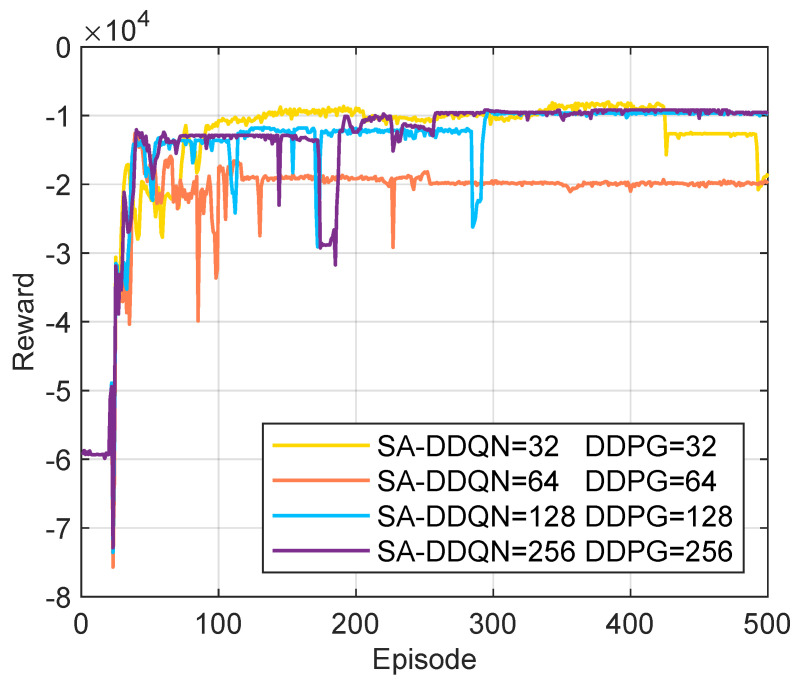
Reward under different batch size parameters.

**Figure 5:**
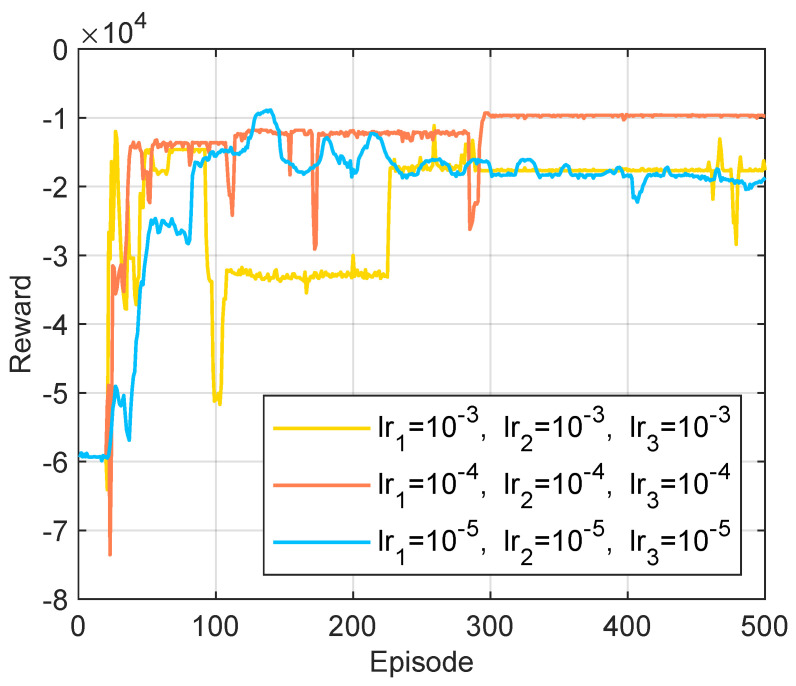
Reward under different learning rate parameters.

Appropriate setting of hyperparameters significantly impacts the performance and learning efficiency of HHDRL algorithms. We conducted a brief sensitivity analysis on two key hyperparameters: batch size and learning rate. Figure 4 shows that when the batch sizes of both SA-DDQN and DDPG are set to 128 or 256, the system achieves stable convergence with similar performance. However, a batch size of 256 significantly slows down training. Therefore, we select 128 as the default value for subsequent simulations. As shown in Figure 5, the system achieves the best reward performance and convergence stability when the learning rates for SA-DDQN and the actor and critic networks of DDPG ($lr_{1}$, $lr_{2}$ and $lr_{3}$) are all set to $10^{-4}$. The learning rate offers a good trade-off between convergence speed and training stability, and is adopted as the default setting in all subsequent experiments.

**Figure 6:**
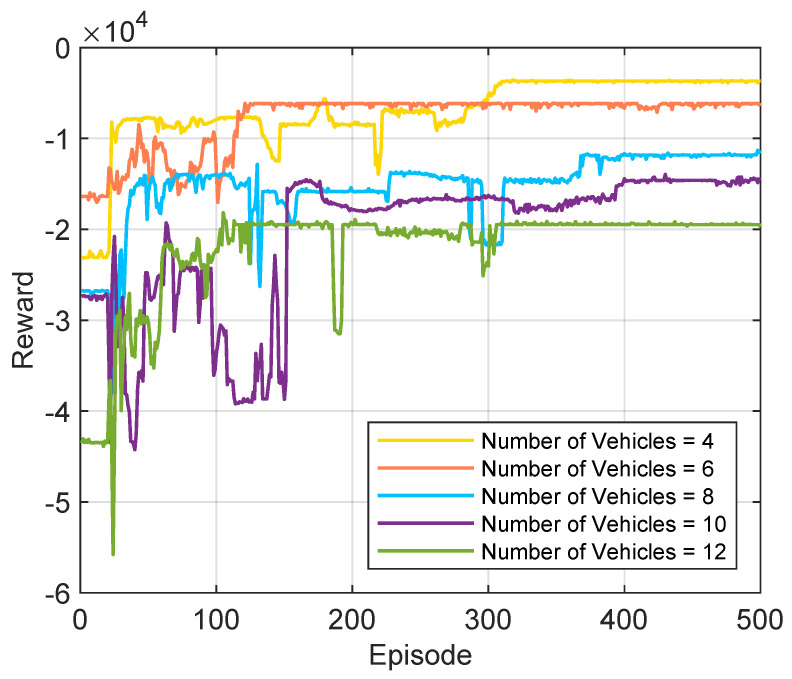
Convergence performance for different ICV numbers.

Figure 6 illustrates the convergence performance of the proposed HHDRL under different numbers of ICVs. It is evident that, for all scenarios, the rewards for each episode gradually increase with the number of training iterations. In the initial stages of the reward curve, there is a rapid upward trend, with some fluctuations along the way, but eventually, it stabilizes. This phenomenon can be attributed to the HHDRL algorithm’s iterative learning process, characterized by continuous trial-and-error learning to refine actions. Once HHDRL achieves effective training, the rewards stabilize at relatively high levels. This indicates that the agent based on the HHDRL algorithm can flexibly and efficiently adapt to changes in the number of vehicles in the environment, providing optimal decisions for task offloading and resource allocation.

**Figure 7:**
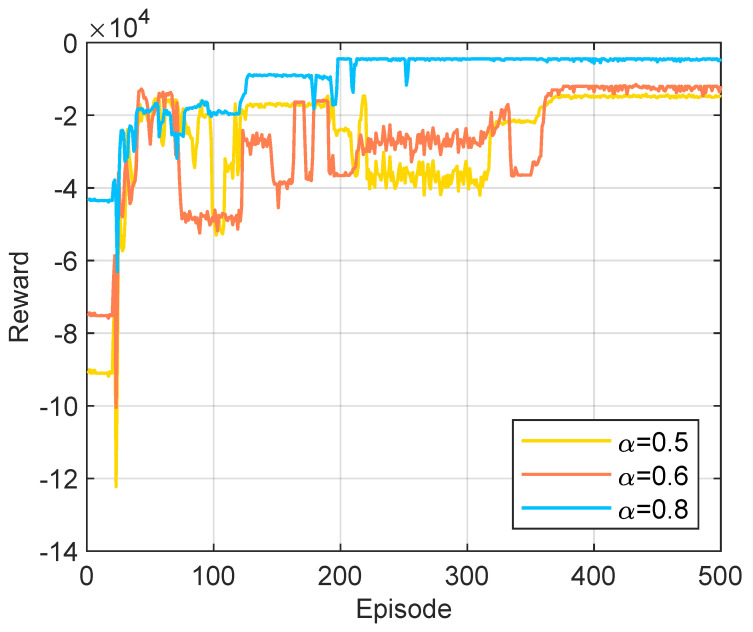
Convergence performance with different values of α.

Figure 7 demonstrates the convergence of the HHDRL algorithm under different values of α. It is observed that in all scenarios, the total reward of the HHDRL algorithm tends to stabilize, indicating good convergence. As α increases, the proportion of task computation time cost in the total cost steadily increases, leading to a gradual decrease in the total cost. This indicates that when emphasizing time cost, the total reward significantly increases, resulting in a reduction in total cost. Conversely, when the focus shifts to energy cost, the total reward decreases noticeably, leading to an increase in total cost. HHDRL can effectively adapt to changes in the proportion between task computation time cost and task computation energy cost, achieving goal balancing. Consequently, users can flexibly set the α value according to their desired objectives. We set α to 0.8 in the next simulation.

### 5.3. Comparative Analysis of Simulation Results

**Figure 8:**
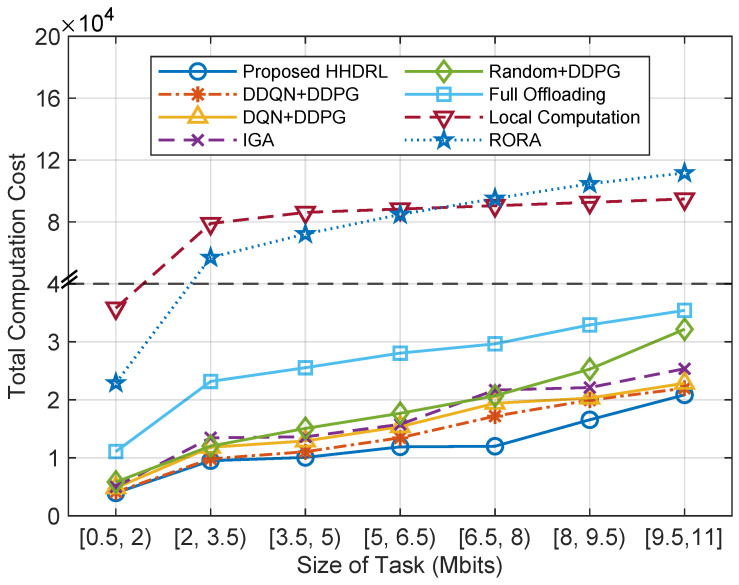
The total computation cost of different algorithms against the size of tasks.

Figure 8 illustrates the comparison of total computation cost across different algorithms under varying task sizes. It can be observed that the total computation cost increases with the growth of task size for all algorithms. However, the proposed HHDRL algorithm consistently achieves the lowest cost in all intervals, demonstrating superior performance in task scheduling optimization and adaptability to increasing computational loads. In contrast, RORA and Local Computation exhibit acceptable performance under small tasks but suffer sharp cost increases as task size grows, due to lack of coordination or limited onboard resources. DDQN+DDPG and DQN+DDPG perform comparably to HHDRL under small tasks but degrade under higher loads. Although the IGA algorithm demonstrates certain improvements over Random+DDPG, its total cost control performance remains inferior to that of DDQN+DDPG, DQN+DDPG, and the proposed HHDRL. These results verify the effectiveness and robustness of HHDRL in dynamic offloading and resource allocation scenarios, especially under high-load conditions.

**Figure 9:**
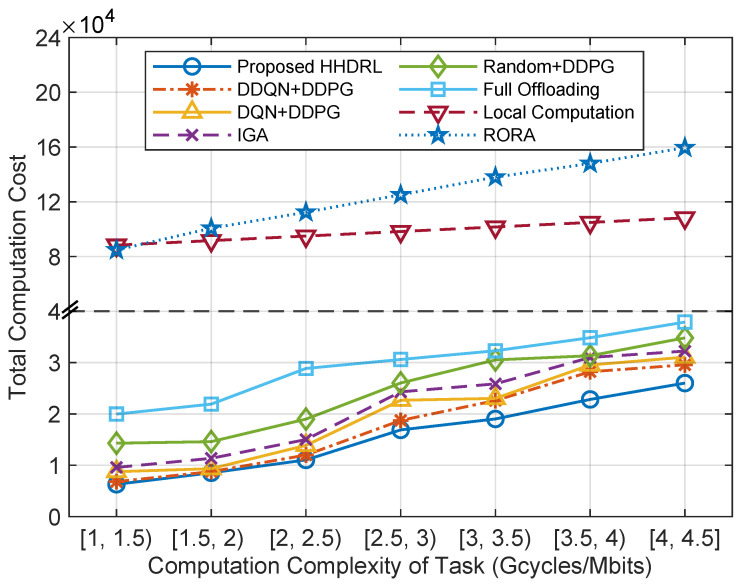
The total computation cost of different algorithms against the computation complexity of tasks.

Figure 9 presents the comparison of the total computation cost for different algorithms under varying task computation complexities. The proposed HHDRL algorithm, overall, outperforms other benchmark algorithms. Both RORA and Local Computation incur high costs, primarily due to the increased local computation costs and noticeable transmission latency increase caused by complexity. For task complexities within interval values such as [1, 1.5), [1.5, 2), and [2, 2.5), the total computational cost of HHDRL is quite close to DDQN+DDPG, DQN+DDPG, and IGA. However, as task complexity increases, the advantage of HHDRL becomes more pronounced. This is because when task complexity is relatively low, both the local processor and MEC are insensitive to variations. As task complexity significantly rises, it leads to an increase in the computational costs of the computing system.

**Figure 10:**
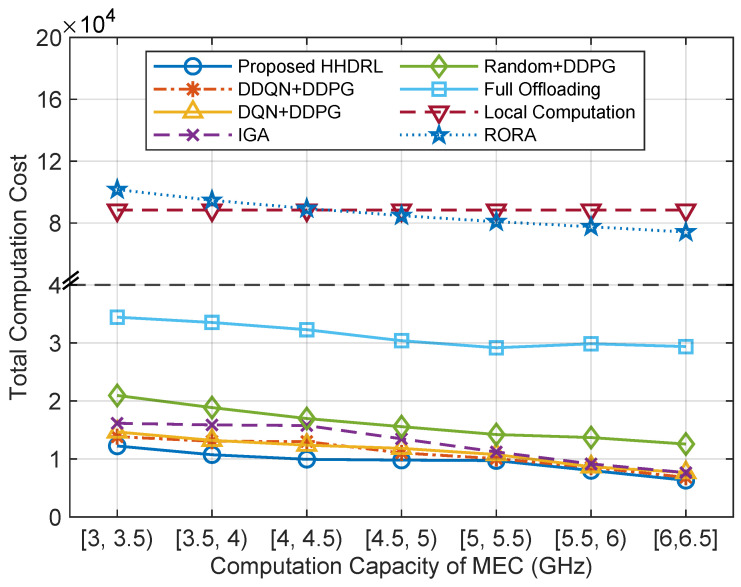
The total computation cost of different algorithms against the computation capacity of MEC servers.

Figure 10 illustrates the comparison of total computation cost across different algorithms under varying MEC server computation capacities. It is evident that as the available computational capacity of MEC servers increases, the computational costs decrease for all algorithms except Local Computation. This reduction is due to the increased computational power of MEC servers, which lowers the task processing latency in MEC. Although the local task computation cost and offloading latency remain constant, the rise in energy cost per unit of time due to MEC is balanced by the decrease in MEC energy cost resulting from the reduced processing latency. Consequently, the overall computational cost is minimally affected. The proposed HHDRL algorithm outperforms other benchmark algorithms, but as the available computational power of MEC continues to increase, the advantage of HHDRL gradually diminishes. In particular, when the computation capacity of MEC servers falls within the ranges of [5, 5.5), [5.5, 6), and [6, 6.5], its performance is only slightly better than that of DDQN+DDPG and DQN+DDPG. This decline occurs because, when MEC computational power reaches a certain level, it can adequately meet the current task demands, rendering excess MEC computational power less significant. The utilization of excess MEC computational power can be enhanced by increasing the number of vehicles and tasks.

**Figure 11:**
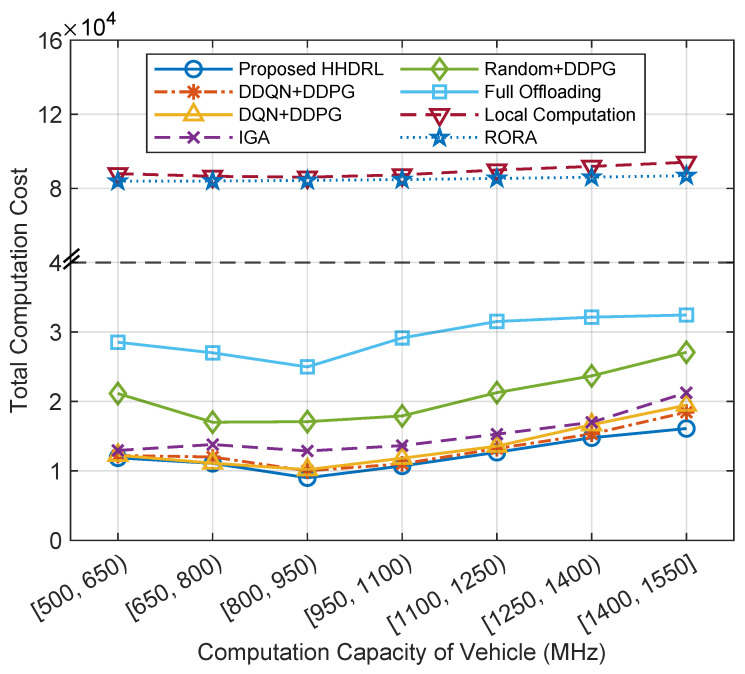
The total computation cost of different algorithms against the computation capacity of vehicles.

Figure 11 illustrates the comparison of total computation cost across different algorithms under varying vehicle computation capacities. It can be observed that as the vehicle’s local computational capacity increases, the RORA algorithm, due to the randomness of its decisions, experiences minimal impact from the increase in local processor computational capacity in terms of the total cost. In contrast, the computational costs of HHDRL, DDQN+DDPG, DQN+DDPG, IGA, Random+DDPG, Full Offloading, and Local Computation initially decrease and then increase. Also, Figure 11 shows that the proposed HHDRL algorithm outperforms other benchmark algorithms. This is because as the vehicle’s local processor computational capacity increases from [500, 650) MHz to [800, 950) MHz, the improvement in local computational capacity reduces the processing latency for tasks handled locally, despite a slight increase in energy consumption per unit of time. However, the decrease in processing latency has a minimal impact on local processing costs. In this experiment, where task size and complexity are fixed, as the vehicle’s local processor computational capacity increases, the total cost increases due to higher local computational energy consumption. Still, given that the vehicle’s local processor computational power is much lower than that of MEC servers, its influence on the overall cost is limited.

**Figure 12:**
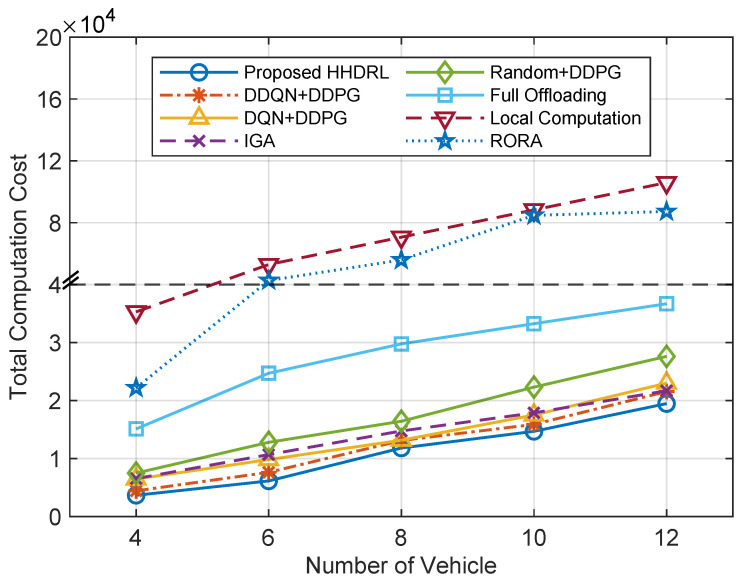
The total computation cost of different algorithms against the number of vehicles.

Figure 12 delineates the comparison of total computation cost across different algorithms under varying numbers of vehicles. It can be observed that as the number of vehicles increases, the total computation cost of all algorithms shows an upward trend. The increase is mainly attributed to the larger overall task volume and intensified communication competition among vehicles, which result in higher execution delay and energy consumption. In contrast, the proposed HHDRL algorithm consistently achieves the lowest total computation cost across all vehicle scales, demonstrating its global optimization capability for task offloading and resource allocation in complex multi-vehicle environments. Especially when the number of vehicles is large (e.g., 10 and 12), other algorithms such as DQN+DDPG, DDQN+DDPG, and RORA experience a much sharper cost increase, whereas HHDRL maintains a relatively low growth rate, indicating its superior scalability and resource scheduling efficiency.

**Figure 13:**
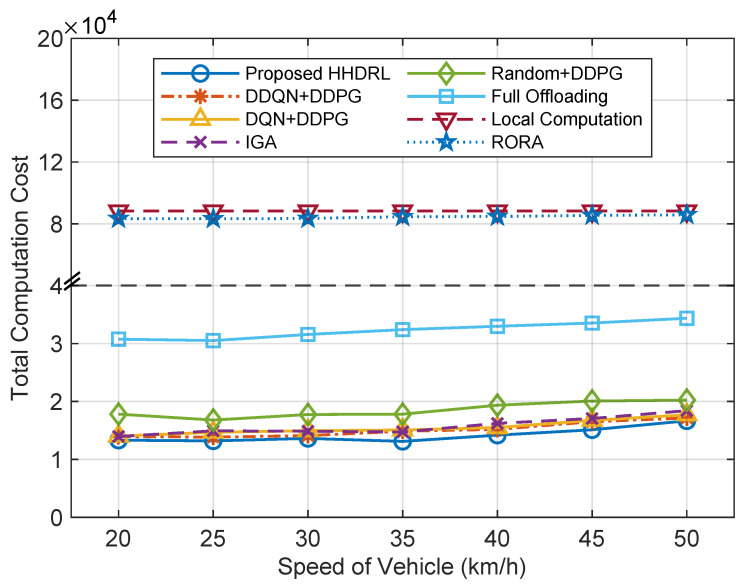
The total computation cost of different algorithms against the speed of vehicles.

Figure 13 depicts the comparison of total computation cost across different algorithms under varying vehicle speeds. It can be observed that under different vehicle speeds, the total computation cost of all algorithms remains generally stable, indicating that speed variation has a relatively small impact on the overall system performance. However, as the vehicle speed increases further, there is a slight increase in the total cost. Compared with other algorithms, the proposed HHDRL algorithm consistently achieves the lowest total computation cost, demonstrating its robustness and adaptability under various speed conditions. Particularly within the range of 30–45 km/h, HHDRL exhibits the smallest performance fluctuation and significantly outperforms baseline methods such as Random+DDPG and Full Offloading, indicating that the algorithm can effectively coordinate task offloading and resource allocation in dynamic communication environments to maintain optimal time and energy cost performance.

Table 2 presents the average decision-making time per time slot over 200 tasks for different algorithms. The proposed HHDRL algorithm achieves near-real-time decision-making efficiency, with an average computation time of $1.013\times 10^{-3}$ s. Although this value is relatively higher than those of other algorithms, it remains negligible relative to the task maximum execution delay of [0.5, 3] s and therefore has minimal impact on overall task completion. In contrast, the decision-making time of IGA reaches 31.58 s, which makes it unsuitable for VECNs requiring strict real-time responses. Other algorithms (including DDQN+DDPG, DQN+DDPG, Random+DDPG, Full Offloading, Local Computation, and RORA) exhibit slight variations in decision-making time, but the overall differences are limited. Considering the task computation cost comparison discussed earlier, the proposed HHDRL not only demonstrates excellent near-real-time performance but also achieves efficient global task scheduling and resource allocation. Therefore, it exhibits superior applicability and overall performance in vehicular edge computing systems.

### 5.4. Ablation Study on Network Structures

**Figure 14:**
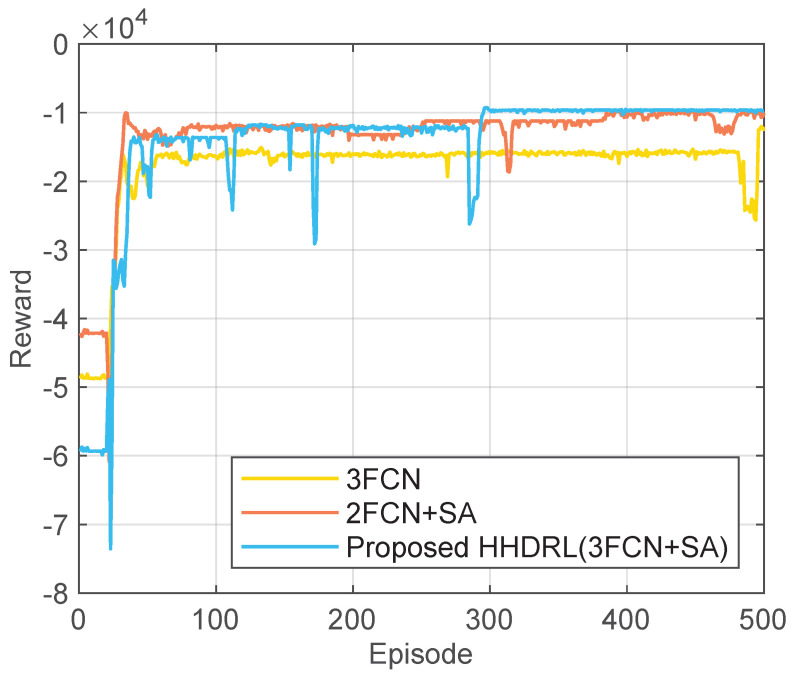
Ablation study of SA-DDQN with different network structures.

To further validate the impact of network structure design in the SA-DDQN, we conducted an ablation study by comparing three different configurations: (i) a three-layer fully connected network (3FCN) without the self-attention layer; (ii) a two-layer fully connected network (2FCN+SA) with the self-attention layer; and (iii) the proposed HHDRL algorithm, namely a three-layer fully connected network enhanced with a self-attention layer (3FCN+SA). The comparison results are illustrated in Figure 14.

As shown in the figure, the proposed HHDRL (3FCN+SA) achieves superior performance in both convergence speed and cumulative rewards compared with the other two variants. Specifically, while 3FCN without the self-attention layer provides relatively stable but limited rewards, 2FCN+SA converges more quickly in the early stages but fails to sustain high long-term performance. By contrast, incorporating the self-attention layer into a three-layer structure enables the model to better capture correlations among input features, highlight critical information, and suppress redundant signals. This design not only accelerates the training process but also yields significant improvements in stability and overall effectiveness. This ablation study demonstrates the effectiveness of incorporating the self-attention mechanism and deeper network structures into the SA-DDQN, highlighting that their combination plays a crucial role in enhancing the overall performance of the proposed HHDRL framework.

## 6. Conclusions

In the paper, we propose a typical vehicle–road collaborative edge computing network. Operating within finite resources and environmental constraints, we aim to minimize the comprehensive cost, which is a weighted combination of time and energy costs, by judiciously scheduling task offloading, allocating transmission power, MEC computational resources, and task offloading ratios. To solve this complex optimization problem, we propose a DRL-based HHDRL algorithm. The upper layer of the algorithm generates the CD as a discrete variable through SA-DDQN, and the lower layer DDPG generates three continuous variables: task offloading ratio, communication power, and MEC computing power allocation ratio. Numerical simulation experiments show that HHDRL has good stability and convergence, and also has good environmental adaptability compared to other benchmark algorithms. In future work, we will investigate multi-agent coordination among ICVs as well as joint task offloading and resource allocation in more complex network topologies.

## Figures and Tables

**Table 1:** Simulation parameters.

Parameter	Value
The length of the road *L*	1800 m
The width of the road *W*	15 m
The width of each lane *H*	3 m
The number of lanes *Q*	4
The coverage radius of each RSU *R*	500 m [40,41,42]
The number of ICVs *N*	[4, 6, 8, 10, 12]
The number of MEC servers *M*	2
The total time duration *T*	8 s
The length of each time slot Δt	0.2 s
The maximum execution delay of the offloading task $w_{i}^{V}\left(t\right)$	[0.5, 3] s [7,46]
The speed of the *i*-th ICV $v_{i}$	[20, 25, 30, 35, 40, 45, 50] km/h
The size of the offloading task $s_{i}^{V}\left(t\right)$	[0.5, 2), [2, 3.5), [3.5, 5), [5, 6.5), [6.5, 8), [8, 9.5), [9.5, 11) Mbits [34,43]
The computational complexity of the offloading task $c_{i}^{V}\left(t\right)$	[1, 1.5), [1.5, 2), [2, 2.5), [2.5, 3), [3, 3.5), [3.5, 4), [4, 4.5) Gcycles/Mbits [46]
The computing capacity of the ICVs $f_{i}^{V}\left(t\right)$	[500, 650), [650, 800)], [800, 950), [950, 1100), [1100, 1250), [1250, 1400), [1400, 1550) MHz [7,43]
The available computing resources of MEC servers $f_{j}^{M}\left(t\right)$	[3, 3.5), [3.5, 4), [4, 4.5), [4.5, 5), [5, 5.5), [5.5, 6), [6, 6.5) GHz [34]
The maximum power of the *j*-th MEC server $p_{j}^{M}\left(t\right)$	90 W
The channel gain value per unit reference distance $\alpha_{0}$	−50 dB [47]
The energy coefficient κ	$10^{-26}$ [25]
The system communication bandwidth *B*	2 MHz [25]
The power of Gaussian white noise $\sigma^{2}$	−100 dBm [47]
The transmission loss $P_{\mathrm{loss}}$	20 dB [47]
The maximum communication transmission power $P_{\max}$	1 W [25]

**Table 2:** Average decision-making time per time slot for different algorithms.

Algorithm	Average Time (s)
Proposed HHDRL	$1.013\times 10^{-3}$
DDQN+DDPG	$9.981\times 10^{-4}$
DQN+DDPG	$9.974\times 10^{-4}$
IGA	31.58
Random+DDPG	$1.496\times 10^{-4}$
Full Offloading	$1.795\times 10^{-4}$
Local Computation	$8.086\times 10^{-6}$
RORA	$6.263\times 10^{-6}$

## Data Availability

Data are available on request due to restrictions.

## Abbreviations

The following abbreviations are used in this manuscript:

ICV	Intelligent connected vehicle
VECN	Vehicular edge computing network
MEC	Mobile edge computing
HHDRL	Hybrid hierarchical deep reinforcement learning
DDQN	Double deep Q-network
DDPG	Deep deterministic policy gradient
IoT	Internet of Things
RSU	Roadside unit
VT	Vehicle terminal
DRL	Deep reinforcement learning
TOSRA	Task offloading scheduling and resource allocation
CD	Communication decision
PTORADs	Power control, task offloading, and resource allocation decisions
GA	Genetic algorithm
V2I	Vehicle-to-infrastructure
SA-DDQN	Self-attention-enhanced DDQN
FCSAN	Fully connected self-attention network
DNN	Deep neural network
DQN	Deep Q-network
